# Characterization of the complete chloroplast genome of the medicinal herb *Eleutherococcus nodiflorus* and its phylogenetic implications

**DOI:** 10.1080/23802359.2022.2116954

**Published:** 2022-10-30

**Authors:** Minjun Li, Qiuyi Gong, Manjia Zhou, Qiang Liu, Rubin Cheng

**Affiliations:** a School of Pharmaceutical Sciences, Zhejiang Chinese Medical University, Hangzhou, China; b Academy of Chinese Medical Science, Zhejiang Chinese Medical University, Hangzhou, China

**Keywords:** *Eleutherococcus nodiflorus*, complete chloroplast genome, diversity regions, phylogenetic analysis, *Eleutherococcus* genus

## Abstract

*Eleutherococcus nodiflorus (Dunn)* S. Y. Hu is a momentous medicinal plant belonging to the Araliaceae family. In the current investigation, we determined the complete chloroplast genome of *E. nodiflorus* and analyzed the phylogenetic relationship among *Eleutherococcus* plants. The chloroplast genome of *E. nodiflorus* exhibited a typical quadripartite structure with a full length of 156,770 bp, including 133 genes, containing 88 protein-coding genes, 8 rRNA genes, 37 tRNA genes, and 1 presumed pseudogene (*ycf1*). The overall GC content observed was 37.95%, with the highest GC content of 43.02% found in the IR region. Comparative genome analysis revealed five highly variable regions among *Eleutherococcus* species, providing potential markers for further investigations on species identification and population genetics. A total of 44 small simple repeats were identified throughout the chloroplast genome of *E. nodiflorus*. The phylogenetic analysis indicated a sister relationship between *E. nodiflorus* and *E. eleutheristylus*, suggesting a close genetic relationship between the two *Eleutherococcus* plants. These results enhance the understanding of the plant evolution within *Eleutherococcus* plants and provide basic genetic resources for the development of species identification and investigation of population genetic diversity of the *Eleutherococcus* genus and Araliaceae.

*Eleutherococcus nodiflorus (Dunn)* S. Y. Hu 1980 is a medicinal plant belonging to the *Eleutherococcus* genus of the Araliaceae family. The *Eleutherococcus* genus was comprised of more than 40 shrub species, which were mainly distributed in eastern Asia. The dried roots and barks of *E. nodiflorus* were used as one of the sources of Chinese medicine Acanthopanacis cortex for expelling wind and dampness, nourishing the liver and kidney, etc. Acanthopanacis cortex was also reported to exhibit anti-aging activities, regulating blood pressure, and lowering blood sugar (Yang et al. 2020). The Acankoreanogenin A from the leaves of *E. nodiflorus* inhibited the release of pro-inflammatory cytokine HMGB1, revealing the potential of a candidate therapy for fulminant hepatitis (Zhang et al. 2011). Due to their similar morphological characteristics, the Acanthopanacis cortex is frequently mistaken with root barks from closely related *Eleutherococcus* species (Wang et al. 2005). The adulterants of Acanthopanacis cortex significantly affected its clinical efficiency and brought potential safety issues (Liang et al. 2014). The complete chloroplast (cp) genomes present a powerful molecular strategy for species identification and phylogenetic relationship investigation at the general and tribe levels (Dong et al. 2021; Zhou et al. 2021). Therefore, determining the complete cp genome of *E. nodiflorus* is fascinating and necessary to contribute to plant authentication and taxonomic classification of the genus *Eleutherococcus*.

Fresh leaf samples of *Eleutherococcus nodiflorus* were collected from the Zhejiang Chinese Medical University’s Botanical Garden of Medicinal Plants in the Fuyang area (30°05′8″ N, 119°52′53″ E). The leaf specimen was submitted at the Medicinal Herbarium Center of the Zhejiang Chinese Medical University (https://yxy.zcmu.edu.cn; Herbarium Code: MHCZCMU; Collector: Rubin Cheng, biothcheng@hotmail.com) under the voucher number XZWJ-20200713. The extracted total genomic DNA was subjected to sequencing using the Illumina Hiseq Platform following our previous reports (He et al. 2021; Wang et al. 2021). To obtain the chloroplast genome of *E. nodiflorus*, the raw reads were trimmed using Trimmomatic and assembled the resulting cleaned reads using metaSPAdes 3.13.0 along with the cp genome of *Eleutherococcus brachypus* (NC_050832) as the reference (Bolger et al. 2014; Nurk et al. 2017). The chloroplast genome of *E. nodiflorus* was annotated with GeSeq and further confirmation was done using BLAST (Tillich et al. 2017). The complete cp genome of *E. nodiflorus* was submitted to GenBank database under accession number MZ362512.

The full-length cp genome of *E. nodiflorus* was 156,770 bp long, with the conserved quadripartite structure consisting of a LSC region (86,710 bp), an SSC region (18,174 bp), and two IR regions of 25,943 bp each. A total of 133 genes were identified in the cp genome of *E. nodiflorus*, containing 88 protein-coding genes, 8 rRNAs, 37 tRNAs, and 1 pseudogene (*ycf1*). It also contained 17 repetitive genes in the IR region, including 7 tRNAs, 4 rRNAs, and 6 protein-coding genes. The most frequently used amino acid in *E. nodiflorus* was Leucine (Leu) (10.58%), followed by Isoleucine (Ile) (8.40%), Serine (Ser) (7.74%), and Glycine (Gly) (6.99%). The tRNA genes in *E. nodiflorus* varied in length between 48 and 93 nucleotides, with the GC content ranging from 41.09% to 62.16%. The comparative genome analysis of *Eleutherococcus* species revealed five diverse regions among the cp genomes with Pi values higher than 0.01, providing potential markers for species identification and phylogenetic investigations of the *Eleutherococcus* genus (Supplementary Material Fig. S1). The *clpP1* was the highest variable gene identified, locating in the LSC region with the Pi value of 0.01867. We also determined 44 small simple repeats in the cp genome of *E. nodiflorus* with length ranging from 10 to 17 bp.

To further explore the phylogenetic relationship of *Eleutherococcus*, the ML tree was constructed using MEGA X. The tree results indicated that *E. nodiflorus* clustered with *E. eleutheristylus* with high support values, suggesting a relatively close genetic relationship between the two *Eleutherococcus* plants (Figure 1). In addition, a core group of five *Eleutherococcus* plants (*E. brachypus*, *E. leucorrhzus*, *E. trifoliatus*, *E. nodiflorus,* and *E. eleutheristylus*) was observed in the ML tree, providing insights for the taxonomic revision in *Eleutherococcus* genus (Figure 1). The nine *Eleutherococcus* species combined together to form a monophyletic group, which displayed a sister relationship with the group of the *Panax* and *Dendropanax*. However, *P. stipuleanatus* and *P. zingiberensis* were divided into two clades in the ML tree, indicating the requirement for further investigations and revisions for the *Panax* genus. These findings provide the basic cp genome information of *E. nodiflorus,* which would contribute to the development of species identification strategies in the future and promote the investigations of population genetics and evolutionary relationships within *Eleutherococcus* and Araliaceae plants.

**Figure 1.**
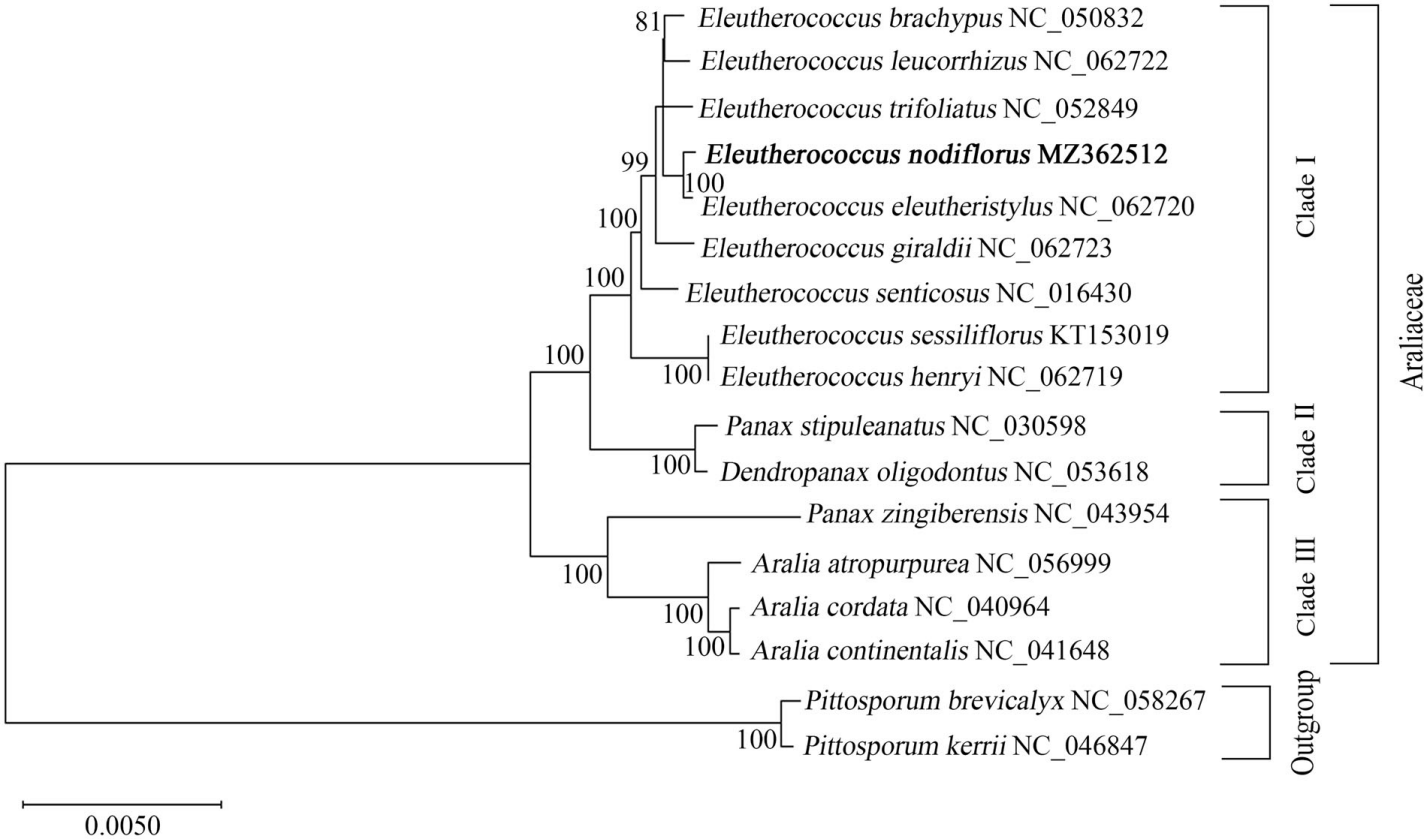
Phylogenetic relationship between newly sequenced *Eleutherococcus nodiflorus* and other representative species of Araliaceae based on complete chloroplast genome analysis. The tree was generated with 71 protein-coding genes of *E. nodiflorus* including the closely related plants in the *Eleutherococcus* and Araliaceae using the maximum likelihood (ML) method by MEGA X. The ML tree was analyzed based on the Kimura 2-parameter model. The *Pittosporum brevicalyx* and *Pittosporum kerrii* were chosen as the outgroup. The newly identified genome of *Eleutherococcus nodiflorus* was represented in bold. Numbers on nodes represented bootstrap values for 81, 99, and 100 replicates in the ML analysis. The GenBank accession number was listed after the species name.

## Supplementary Material

Supplemental Material

## Data Availability

The genome sequence data that support the findings of this study are openly available in GenBank of NCBI at (https://www.ncbi.nlm.nih.gov/) under the accession number MZ362512. The associated BioProject, SRA, and BioSample numbers of *E. nodiflorus* are PRJNA808818, SRR18086324 and SAMN26107482, respectively.
