# Supplementary material for: Characterization of the complete chloroplast genome of the medicinal herb Eleutherococcus nodiflorus and its phylogenetic implications
Source: Mitochondrial DNA B Resour. 2022 Oct 30;7(11):1890–2. doi: 10.1080/23802359.2022.2116954 (PMC9629086; doi:10.1080/23802359.2022.2116954)

Supplementary Figure

Characterization of the complete chloroplast genome of the medicinal herb *Eleutherococcus nodiflorus* and its phylogenetic implications

Minjun Li[1], Qiuyi Gong[1], Manjia Zhou[1], Qiang Liu[1], Rubin Cheng[1,2] *

[1] School of Pharmaceutical Sciences, Zhejiang Chinese Medical University, Hangzhou, China

[2] Academy of Chinese Medical Science, Zhejiang Chinese Medical University

*Correspondence: Rubin Cheng

Figure S1

Nucleotide diversity (Pi) in the cp chloroplast genomes of *Eleutherococcus nodiflorus* and other 4 species from tribe Araliaceae (*Eleutherococcus trifoliatus*, *Eleutherococcus brachypus*, *Eleutherococcus senticosus*, *Eleutherococcus sessiliflorus*). Window length = 600bp; Step size = 200bp. The detailed location of the midpoint was displayed at the x-axis, while the nucleotide diversity of each window was presented at the Y-axis.

Figure S1

Nucleotide diversity (Pi) in the cp chloroplast genomes of *Eleutherococcus nodiflorus* and other 4 species from tribe Araliaceae.

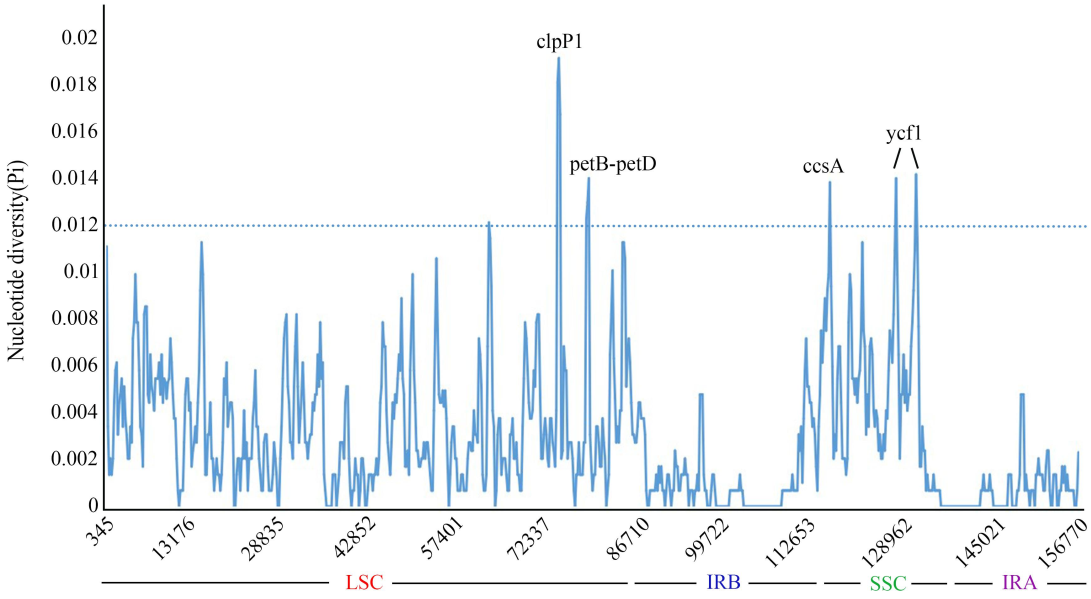

Supplement: Supplemental Material [file TMDN_A_2116954_SM5916.pdf]
